# *Bacillus* spp. Isolated from Puba as a Source of Biosurfactants and Antimicrobial Lipopeptides

**DOI:** 10.3389/fmicb.2017.00061

**Published:** 2017-01-31

**Authors:** Karla J. Perez, Jaime dos Santos Viana, Fernanda C. Lopes, Jamile Q. Pereira, Daniel M. dos Santos, Jamil S. Oliveira, Renata V. Velho, Silvia M. Crispim, Jacques R. Nicoli, Adriano Brandelli, Regina M. D. Nardi

**Affiliations:** ^1^Laboratório de Microbiologia Aplicada, Departamento de Microbiologia, Instituto de Ciências Biológicas, Universidade Federal de Minas GeraisBelo Horizonte, Brazil; ^2^Laboratório de Bioquímica e Microbiologia Aplicada, Departamento de Ciência de Alimentos, Instituto de Ciência e Tecnologia de Alimentos, Universidade Federal do Rio Grande do SulPorto Alegre, Brazil; ^3^Núcleo de Biomoléculas, Departamento de Bioquímica-Imunologia, Instituto de Ciências Biológicas, Universidade Federal de Minas GeraisBelo Horizonte, Brazil

**Keywords:** antimicrobial peptides, biosurfactant, fermented food, *Bacillus* spp., cassava, MALDI-TOF mass spectrometry

## Abstract

Several products of industrial interest are produced by *Bacillus*, including enzymes, antibiotics, amino acids, insecticides, biosurfactants and bacteriocins. This study aimed to investigate the potential of two bacterial isolates (P5 and C3) from puba, a regional fermentation product from cassava, to produce multiple substances with antimicrobial and surface active properties. Phylogenetic analyses showed close relation of isolates P5 and C3 with *Bacillus amyloliquefaciens* and *Bacillus thuringiensis*, respectively. Notably, *Bacillus* sp. P5 showed antimicrobial activity against pathogens such as *Listeria monocytogenes* and *Bacillus cereus*, in addition to antifungal activity. The presence of genes encoding pre-subtilosin (*sboA*), malonyl CoA transacylase *(ituD)*, and the putative transcriptional terminator of surfactin (*sfp*) were detected in *Bacillus* sp. P5, suggesting the production of the bacteriocin subtilosin A and the lipopeptides iturin A and surfactin by this strain. For *Bacillus* sp. C3 the presence of *sboA* and *spas* (subtilin) genes was observed by the first time in members of *B. cereus* cluster. *Bacillus* sp. P5 showed emulsifying capability on mineral oil, soybean biodiesel and toluene, while *Bacillus* sp. C3 showed emulsifying capability only on mineral oil. The reduction of the surface tension in culture medium was also observed for strain P5, confirming the production of surface-active compounds by this bacterium. Monoprotonated molecular species and adducts of sodium and potassium ions of surfactin, iturin, and fengycin were detected in the P5 culture medium. Comparative MS/MS spectra of the peak *m/z* 1030 (C14 surfactin A or C15 surfactin B [M+Na]^+^) and peak *m/z* 1079 (C15 iturin [M+Na]^+^) showed the same fragmentation profile of standards, confirming the molecular identification. In conclusion, *Bacillus* sp. P5 showed the best potential for the production of antifungal, antibacterial, and biosurfactant substances.

## Introduction

Spontaneous fermented foods are sources of microorganisms that frequently produce antimicrobial molecules. Puba or carimã is a Brazilian staple food made by spontaneous submerged fermentation of cassava (*Manihot esculenta*, Crantz) roots (Crispim et al., 2013). Traditional fermentation of cassava is dominated by lactic acid bacteria, but yeast and *Bacillus* spp. were also described Lacerda et al. (2005). *Bacillus* spp. are known as cassava endophytic bacteria (Melo et al., 2009). Members of the genus *Bacillus* are considered good producers of molecules with antimicrobial activity. Among the useful metabolites produced by *Bacillus* spp., a diversity of peptide antibiotics has been described by Stein (2005). These include well known substances such as bacitracin, bacteriocins and antimicrobial lipopeptides produced by multiple-step enzymatic processes (Stein, 2008; Abriouel et al., 2011). Moreover, some lipopeptides produced by *Bacillus* are biosurfactants of great interest, given that they may be explored as an alternative to synthetic surfactants, providing advantages such as biodegradability and low toxicity to humans, animals, and plants (Angelini et al., 2009).

*Bacillus* strains produce lipopeptides that can be divided into three major families: surfactins, iturins and fengycins or plispastatins. Surfactins and iturins are composed by cyclic heptapeptides, which contain a β-hydroxy fatty acid and β-amino fatty acid, respectively (Bonmatin et al., 2003; Ongena and Jacques, 2008). Surfactin, besides the antimicrobial activity, shows an outstanding surface-active property (Chen et al., 2008; Shaligram and Singhal, 2010). Iturins are a family of lipopeptides that present remarkable antifungal activity (Chen et al., 2008; Hsieh et al., 2008), while fengycin is a cyclic decapeptide with a β-hydroxy fatty acid in its side chain (Wu et al., 2007). These lipopeptides differ one from each other in the length and branching of the fatty acid side chains and the amino acid substitutions in the peptide ring (Ongena and Jacques, 2008).

Several strains of *Bacillus subtilis* and *Bacillus amyloliquefaciens* have been described to produce different antimicrobial lipopeptides. In response to nutritional stress, a variety of processes are activated in *Bacillus* strains, including sporulation, synthesis of extracellular degradative enzymes and antibiotic production (Stein, 2005; Caldeira et al., 2011). Moreover, some lipopeptides have potential for agricultural and environmental applications, including the antagonistic activity against a wide range of phytopathogens, and the promotion of host defense mechanisms through beneficial interaction of *Bacillus* species with plants (Ongena and Jacques, 2008). Some lipopeptides from *Bacillus* spp. are promising as antitumor, antiviral and antimycoplasma agents as well (Yang et al., 2006; Silva et al., 2014). Because of these characteristics, the antimicrobial peptides produced by *Bacillus* are products of interest for food, pharmaceutical and biomedical applications.

This study aimed to search for *Bacillus* strains that could produce multiple bioactive molecules, such as biosurfactants and antimicrobial peptides, among bacterial isolates that are part of puba microbiota. Two strains, namely *Bacillus* sp. C3 and P5, were selected and characterized. Genes related to the production of antimicrobial substances were identified by PCR and sequencing, showing by the first time the presence of genes for the bacteriocins subtilosin A and subtilin in a member of *Bacillus cereus* group, and further MALDI-TOF analyses were performed for characterizing bioactive compounds.

## Materials and Methods

### Microorganisms

The strains *Bacillus* sp. C3 and P5 were isolated from puba as described below. The maintenance of bacterial strains was performed in Brain Heart Infusion broth (BHI, Difco, Sparks, NV, USA) containing 20% (v/v) glycerol at -20°C. The indicator strains used for evaluation of antibacterial and antifungal activity were selected for their importance as human and animal pathogens or relevance as food spoilage agents. The strains are listed in **Table 1**.

**Table 1:** Antimicrobial activity of culture supernatants from *Bacillus* sp. C3 and *Bacillus* sp. P5 against indicator microorganisms.

				Inhibition diameter (mm)	
Indicator microorganism^a^	Source	Culture media	T (°C)	*Bacillus* sp. C3	*Bacillus* sp. P5
**Gram-positive**					
*Bacillus amyloliquefaciens*	LBM 5006	BHI	37	NT	6 ± 0.6
*Bacillus amyloliquefaciens*	ATCC 23350	BHI	37	–	11 ± 0.5
*Bacillus cereus*	ATCC 14579	BHI	37	12 ± 0.4^a^	14 ± 0.5
*Bacillus cereus* A-1	Puba	BHI	37	–	11 ± 0.1^a^
*Bacillus cereus* B-2	Puba	BHI	37	–	10 ± 0.8
*Bacillus cereus* D1	Puba	BHI	37	10 ± 0.5^a^	12 ± 0.5
*Bacillus subtilis*	ATCC 6633	BHI	37	–	10 ± 0.6
*Bacillus subtilis*	DSM 3258	BHI	37	–	13 ± 0.7
*Bacillus subtilis*	ATCC 21228	BHI	37	–	13 ± 0.1
*Bacillus subtilis*	ATCC 7971	BHI	37	–	11 ± 0.2^a^
*Bacillus subtilis*	Food	BHI	37	NT	6 ± 0.4
*Corynebacterium fimi*	NTCS 7547	BHI	37	–	10 ± 0.5
*Lactobacillus acidophilus*	ATCC 4356	MRS	35	NT	2 ± 0.0
*Lactobacillus fermentum*	ATCC 9338	MRS	35	–	–
*Lactobacillus murinus L2*	Mouse	MRS	35	NT	5 ± 0.3
*Listeria monocytogenes*	ATCC 6477	BHI	37	–	12 ± 0.5
*Listeria monocytogenes*	ATCC 15113	BHI	37	–	11 ± 0.2
*Listeria monocytogenes*	ATCC 19112	BHI	37	–	9 ± 0.4
*Listeria monocytogenes*	ATCC 19115	BHI	37	–	8 ± 0.2
*Listeria innocua*	Food	BHI	37	NT	11 ± 0.5
*Staphylococcus aureus*	ATCC 25923	BHI	37	–	–
*Staphylococcus haemolyticus*	Clinical	BHI	37	NT	15 ± 1.5
*Staphylococcus saprophyticus*	Clinical	BHI	37	NT	14 ± 1.0
**Gram-negative**					
*Enterobacter aerogenes*	Food	BHI	37	NT	8 ± 0.8
*Escherichia coli*	ATCC 25922	BHI	37	–	–
*Salmonella* Enteritidis	ATCC 13076	BHI	37	–	–
*Salmonella* Typhimurium	ATCC 13311	BHI	37	–	–
**Filamentous fungi and yeast**					
*Aspergillus flavus*	Food	PDA	30	+	+
*Aspergillus flavus*	Food	PDA	30	–	7 ± 0.5
*Aspergillus fumigatus*	Environmental	PDA	30	–	7.5 ± 0.8
*Aspergillus niger*	Food	PDA	30	+	+
*Fusarium oxysporum* f. sp. *lycopersici*	Environmental	PDA	30	–	80 ± 0.5
*Candida tropicalis*	Clinical	PDA	30	–	10 ± 1.0
*Weissella paramesenteroides*	Food	PDA	30	–	–

*(–) no inhibition; (+) reduced sporulation; NT, not tested. ^a^Halo with partial inhibition.*

### Isolation and Presumptive Identification

Bacterial strains were isolated from samples of puba that were obtained from different batches of the same producer. The samples were obtained in the District of Saco da Raiz in Estância, a town of Sergipe State, located in northeast of Brazil. The strains were selected among Gram-positive rods obtained from puba samples, where five isolates were presumptively identified as *Bacillus* species based on standardized methods including observation of cell morphology Gram-staining, phase-contrast microscopy for detection of parasporal crystal proteins formation, and catalase activity (Food and Drug Administration-Bacteriological Analytical Manual [FDA-BAM], 2012). Additional tests were conducted using the identification kits API 50 CHB and API 20E (BioMérieux SA, Marcy-l’Étoile, France). The results were analyzed by the API LAB Plus software for strain identification (BioMérieux SA). Following, the antimicrobial potential of these strains was tested against varied bacteria, yeasts and filamentous fungi (data not show). The strains C3 and P5, which presented the most promising results, were selected for the subsequent tests.

### Bacterial Identification by Fatty Acid Methyl Ester (FAME) Analysis

Total cellular fatty acids from the isolates *Bacillus* sp. P5 and C3 were analyzed using the MIDI Sherlock^®^ Microbial Identification System (Microbial Identification System, Microbial ID Inc., Newark, NJ, USA). Fatty acid extraction and methyl ester generation were performed with Instant FAME Method kit according to the manufacturer’s instruction. Gas chromatography (GC) was performed on an Agilent 6890N analyzer using calibration standards (*#*1300-AA; MIDI, Inc.). The Sherlock^®^ Microbial Identification (MIDI, Inc., version 4.5) software was used to assign GC peaks to individual fatty acid structures. The identification was made by comparative fatty acids with database to Instant Environmental TSA library (ITSA1) version 1.10. Samples with a similarity index (SI) ≥ 0.5 were considered as an acceptable FAME identification (Kunitsky et al., 2006).

### Phylogenetic Characterization

Total DNA from *Bacillus* sp. C3 and P5 was extracted from overnight cultures of strains using the Promega Wizard SV Genomic DNA kit (Promega, USA) and the amplification of 16S rRNA gene was performed using the universal primers 27F (5′-GAGTTTGATCCTGGCTCAG-3′) and 1525R (5′-AGAAAGGAGGTGATCCAGC C-3′), according to Lisbôa (2006). The PCR conditions were: initial denaturation for 5 min at 95°C, 30 cycles of 30 s at 95°C for denaturation, 1 min 30 s at 46°C for annealing, 80 s at 72°C for extension and 7 min at 72°C for final extension (adapted from Horisawa et al., 2009). The amplicons were sequenced by the ATCGene Laboratory (Porto Alegre, Brazil). The sequences obtained were submitted to the BLAST search algorithm^1^, edited using Bioedit software and aligned with Clustal X. For the construction of the dendrogram, sequences were checked for quality, aligned and analyzed using the software Phred v.0.20425 (Ewing and Green, 1998), Phrap v.0.900319 (Gordon et al., 2001) and Consed 12.0 (Gordon et al., 1998). The phylogenetic tree was developed using the Neighbor-joining method present in MEGA version 5.0 (Kumar et al., 2004). Genetic distance was calculated based on Kimura two-parameter model of nucleotide evolution. The support of nodes was estimated using 1000 bootstrap replicates. The sequences obtained were deposited in GenBank under the accession numbers JX456531 for *Bacillus* sp. C3 and JX456530 for *Bacillus* sp. P5.

### Detection of Putative Genes for Surfactin, Iturin A, Subtilosin A and Subtilin

Gene amplification of *sfp, ituD, sboA*, and *spaS* was performed by PCR with specific primers, as described by Hsieh et al. (2008) and Velho et al. (2011). The following parameters were used: for *ituD* (iturin A), denaturation at 94°C for 1 min., annealing for 1 min at 50°C, elongation at 1.5 min at 72°C, in a total of 30 cycles; for *sfp* (surfactin), denaturation for 1 min at 94°C, annealing for 30 s at 46°C for 1 min and elongation at 72°C for a total of 25 cycles. For *sboA* (subtilosin A), the following parameters were used: 1 min denaturation at 94°C, annealing for 30 s at 50°C for 1 min and elongation at 72°C for a total of 35 cycles; for *spaS* (subtilin), 1 min denaturation at 94°C, annealing for 30 s at 55°C for 1 min and elongation at 72°C for a total of 35 cycles. The amplified products were sequenced by ATCGene Laboratory (Porto Alegre, Brazil). The sequences obtained were submitted to the BLAST search algorithm^2^ and edited using Bioedit software for contigs assembly.

### Production of Antimicrobial Compounds

Erlenmeyer flasks of 125 ml containing 30 ml of BHI broth were inoculated with a loop of *Bacillus* strains cultivated on BHI agar. The inoculum was pre-incubated for 24 h at 125 rpm and 37°C. An aliquot of 1% (v/v) of this culture was transferred to a 500 ml Erlenmeyer flask containing 200 ml of BHI broth. The culture was incubated for 48 h at 42°C with constant stirring at 125 rpm. After this period, the culture was centrifuged for 15 min at 10,000 *g*. The supernatant was sterilized by filtration through a cellulose filter with a pore size of 0.22 μm (for small volumes) or by vacuum filtration through a 0.22 μm silica filter (for larger volumes). The filtrates were kept at 4°C.

### Antimicrobial Activity

The antimicrobial activity of crude supernatants was detected by a modified diffusion assay (Kimura et al., 1998; Motta and Brandelli, 2002). Aliquots (20 μl) of the crude supernatants of *Bacillus* sp. C3 and *Bacillus* sp. P5 were applied onto BHI agar plates previously inoculated with a cell suspension (10^8^ colony forming units CFU/ml – corresponding to 0.5 of McFarland scale) of the indicator microorganism. Zones of inhibition were measured after incubation for 24–48 h under optimal growth conditions for the indicator strain. The inhibitory zones were measured with a digital pachymeter. The test was performed in duplicate with two supernatants obtained from different culture. For this assay, the indicator strains are shown in **Table 1**.

### Detection of Antifungal Activity

The fungal strains selected as indicators for this experiment were *Fusarium oxysporum* f. sp. *lycopersici, Aspergillus fumigatus, Aspergillus flavus, Aspergillus niger*, and *Candida tropicalis* (**Table 1**). They were inoculated on potato dextrose agar (PDA) plates and incubated for 72 h at 30°C. Spore suspensions were prepared for each fungus according to Lopes et al. (2011), with the exception of the yeast *C. tropicalis*, which was prepared by the same method used for the antibacterial activity assay. After the preparation of the suspensions, a final concentration of 10^6^ spores/ml was mixed with melted PDA at 45°C. Then, 15 μl of the filtrates were added, and the plates were incubated at 30°C for 48 h (Rouse et al., 2008). The tests were performed in triplicate.

### Biosurfactant Activity

The method used by Hsieh et al. (2004) was modified as follows. *Bacillus* sp. C3 and *Bacillus* sp. P5 were cultivated for 24 h in tryptic soy broth medium (TSB, Difco) at 37°C and inoculated later in Tryptic casein Soy Agar (TSA, Difco) plates containing 5% (v/v) sheep blood and incubated at 37°C for 24 h. The presence of a zone of hemolysis around the colony was observed for strains producing a biosurfactant.

### Emulsifying Activity

The evaluation of emulsification (Cooper and Goldenberg, 1987) was performed using cultures and growth culture supernatants of *Bacillus* sp. C3 and *Bacillus* sp. P5 grown in BHI medium at 37°C for 24 h. For this test, 2 ml of culture or growth culture supernatant were mixed with 3 ml of hydrophobic compounds (mineral oil, xylene, toluene or soybean biodiesel) in test tubes with flat bottom (100 mm × 15 mm), the mixture was vortexed for 2 min and the flasks left to stand for 24 h. After this period, the emulsifying index was calculated by the following formula (Velho et al., 2011):

$$\begin{array}{c} E_{24}=(H_{e})\times 100/(H_{t}) \end{array}$$

where E_24_ = emulsification index, *H*_e_ = height of the emulsified column, *H*_t_ = total height.

### Surface Tension

The surface tension was measured in the absence of microbial cells, which were removed by centrifugation at 10,000 *g* for 15 min. The samples were maintained for 30 min at room temperature and surface tension was determined using a digital tensiometer (Gibertini, Milan, Italy) using the Wilhelmy plate method (Biswas et al., 2001). Distilled water (72 mN m^-1^) and ethanol (24 mN m^-1^) were used as standards (Cerqueira et al., 2012).

### Extraction of Lipopeptides and Bacteriocins

Cell-free supernatants were processed in two different ways: lipopeptides were isolated by a combination of acid precipitation and solvent extraction procedure following Cooper et al. (1981) and Vater et al. (2002), and bacteriocin were extracted with *n*-butanol as described by Kawulka et al. (2004). In brief, cells were removed from the 6, 30, and 36 h growing culture in BHI broth by centrifugation (13,000 *g*) for 15 min at 4°C. For the acid extraction the supernatant was adjusted to pH 2.0 by addition of HCl and allowed to precipitate at 4°C for 16 h. Precipitate was collected after centrifugation (13,000 *g*) for 20 min at 4°C and extracted with dichloromethane. The lipopeptide containing dichloromethane fraction was collected after filtration and vacuum-dried. Alternatively, the supernatant was extracted by adding one-quarter the volume of *n*-butanol, shaked for 1 h, and then poured into a separator funnel and allowed to stand overnight. The organic layer was separated, concentrated under vacuum and the residue suspended in methanol (10 ml per liter of cell culture) (Kawulka et al., 2004).

### Extraction of Lipopeptides from Puba

Twenty grams of puba were weighted and suspended in 40 ml of distilled water and homogenized. Then, this suspension was submitted to extraction with *n*-butanol as described previously. Zip Tip^®^ pipette was standardized with 10 μl acetonitrile, 10 μl1:1 (v/v) acetonitrile/0.1% trifluoroacetic acid (TFA) and twice with 10 μl of 0.1% (v/v) TFA. The solution with the lipopeptides extracted from puba was loaded to the pipette and the eluate was discarded. The retained material was washed three times with 10 μl of 0.1% (v/v) TFA and the sample was eluted with 5 μl of acetonitrile/0.1% TFA (60:40, v/v). The eluate was analyzed by mass spectrometry.

### Reversed Phase HPLC

The samples were dissolved in methanol and fractionated by reversed phase chromatography. High-performance liquid chromatography (HPLC) was performed on a Shimadzu Prominence equipment (Department of Biochemistry, Immunology, Institute of Biological Sciences, Federal University of Minas Gerais) with a SPD-20A-UV/VIS detector. The column was a Sephasil^TM^ Peptide C18 (5 μm ST 4.6/250 C18, 100-Å pore size, 5 μm particle size). The elution condition was: 0–10 min mobile phase A (0.05% TFA); 10–40 min a gradient of 0–100% mobile phase B (acetonitrile + 0.05% TFA); and 40–50 min mobile phase B. A flow rate of 1 ml/min was used. Column eﬄuent was monitored at 220 and 280 nm, and fractions were checked for antimicrobial activity against *L. monocytogenes* ATCC 7644. The active peaks were selected for further study, including chemical and mass spectrometry analyses. All HPLC solvents were prepared fresh daily and filtered under vacuum before use. All aqueous solutions were prepared with ultrapure water.

### MALDI-TOF Mass Spectrometry Analyses

Active fractions against *L. monocytogenes* ATCC 7644 were freeze-dried and suspended in 50 μl of ultrapure water, and then 0.7 μl of this solution was mixed with 0.7 μl of a saturate matrix solution of α-cyano-4-hydroxy-cinnamic acid (Sigma-Aldrich, St Louis, MO, USA) or 2-5-dihydrobenzoic acid (DHB, Fluka). The matrix solutions were prepared in 1:1 (v/v) CH_3_CN:H_2_O containing 0.1% TFA. The mixtures were spotted onto a MALDI-TOF sample plate (Bruker Daltonics, Inc., Department of Biochemistry, Immunology, Institute of Biological Sciences, Federal University of Minas Gerais), at room temperature. Analysis was performed in a mass spectrometer by matrix-assisted laser desorption ionization time-of-flight mass spectrometry (MALDI-TOF, AUTOFLEX III, Bruker Daltonics) in the positive reflective mode, using the Flex Control 3.3 software (Bruker Daltonics, Inc.). The calibration was performed using Peptide Calibration Standard II (Bruker Daltonics, Inc.). MALDI-MS/MS peptide fragmentation patterns were compared using commercial standards of surfactin and iturin A (Sigma–Aldrich).

## Results

### Characterization of *Bacillus* Strains Isolated from Puba

The isolates P5 and C3 showed cellular morphology typical of spore-forming Gram-positive bacteria and were positive for catalase test and motility, and negative to rhizoid growth. The ability of strain C3 for parasporal crystal protein formation was confirmed by phase contrast microscopy examination (Supplementary Figure S1). The API 50CHB and 20E tests showed 99.1% ID and 0.56 T for strain C3 with *Bacillus cereus*/*thuringiensis*. For strain P5 API test showed 99.8% ID and 0.59 T for *Bacillus subtilis*/*amyloliquefaciens*.

Similarity index values of 0.675 and 0.563 were calculated for *Bacillus thuringiensis*-GC subgroup A and *B. cereus* GC subgroup A, respectively, based on MIDI-FAME profile of *Bacillus* sp. C3. According to the criterion established by FAME analysis, when SI is larger than 0.5 and separated from other organisms from the library by at least 0.100, the isolate is considered identified, in this case as *B. thuringiensis*. For *Bacillus* sp. P5 FAME analyses revealed a SI of 0.452 for *B. subtilis* GC subgroup A and 0.407 for *B. subtilis* subsp. *spizizenii*. When the test was repeated, the SI values were 0.463 for *B. subtilis* subsp. *spizizenii* and 0.458 to *B. subtilis* GC subgroup A. In this case, the SI values were lower than 0.5 and this isolate could not be reliably identified at the species level by MIDI-FAME analysis.

### Phylogenetic Analysis

**FIGURE 1:**
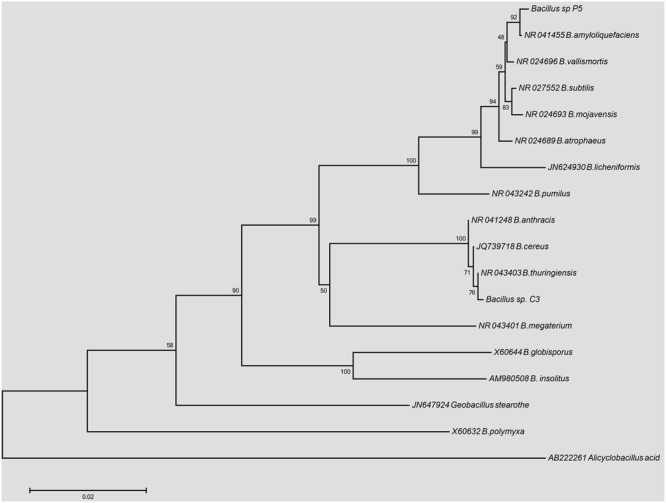
**Phylogenetic tree based on 16S rDNA gene sequences from *Bacillus* sp. C3 and *Bacillus* sp. P5 using the Neighbor-joining method (1000 bootstrap replicates)**.

The phylogenetic reconstruction of the 16S rDNA sequences is shown in **Figure 1**. The strain *Bacillus* sp. P5 was recovered in a node with 92% of support with *B. amyloliquefaciens*, while the strain *Bacillus* sp. C3 was clustered together with the members of *B. cereus* group, with a similarity of 100%, showing a major identity (76%) with *B. thuringiensis.*

### Antimicrobial Activity

The crude supernatants of *Bacillus* sp. C3 and *Bacillus* sp. P5 were tested for antimicrobial activity against Gram-positive and Gram-negative bacteria and fungi. The inhibitory activity of *Bacillus* sp. P5 was observed against most of the bacteria, including major pathogens and food spoilage organisms such as *Bacillus cereus, Listeria monocytogenes, Staphylococcus haemolyticus*, and *Staphylococcus saprophyticus*, as well as against filamentous fungi and the yeast *Candida tropicalis*. On contrast, *Bacillus* sp. C3 showed a narrow spectrum of antimicrobial activity (**Table 1**).

**FIGURE 2:**
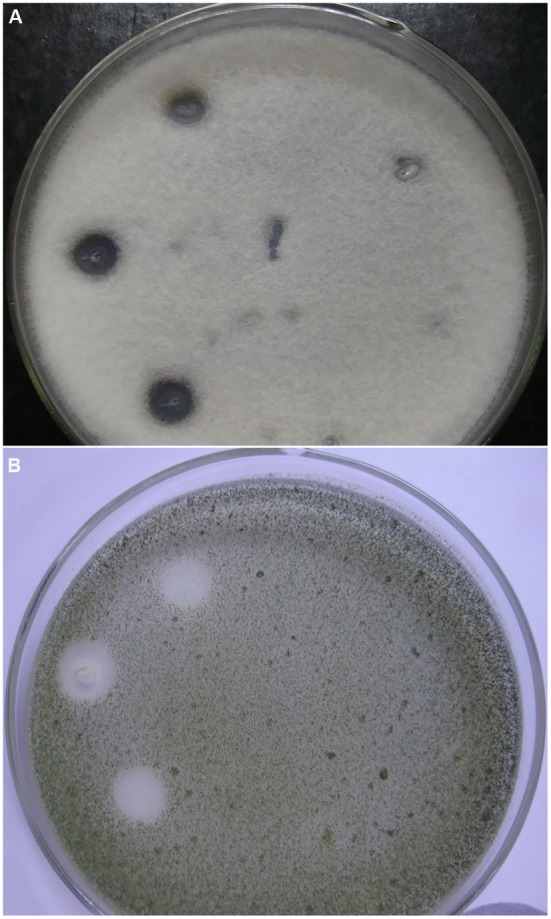
**Antifungal activity of *Bacillus* strains isolated from puba.** Strains were inoculated onto the surface of PDA agar plates containing the filamentous fungi **(A)** *Fusariumoxysporum* f. sp. *lycopersici* and **(B)** *Aspergillus flavus*. Plates show three inoculations of *Bacillus* sp. P5 on the left side and *Bacillus* sp. C3 on the right side.

The antifungal activity of *Bacillus* sp. P5 was notorious, and clear inhibitory halos were observed against filamentous fungi growing onto PDA agar plates (**Figure 2**). *Bacillus* sp. P5 showed a broad spectrum of antifungal activity, inhibiting the growth of all the fungi when tested as whole culture and three of five strains when it was tested as culture supernatant (data not shown).

### Presence of Surfactin, Iturin A, Subtilosin A and Subtilin Genes

PCR assays followed by sequencing were conducted to investigate the occurrence of essential genes for production of antimicrobial peptides. The genes of iturin A (*ituD*), surfactin transcriptional terminator (*sfp*), and subtilosin A (*sboA*), all with 99% of coverage and identity, were detected for *Bacillus* sp. P5 (**Table 2**). For *Bacillus* sp. C3, the identification of the genes encoding subtilosin A (*sboA*) and subtilin (*spaS*) was possible with 99 and 100% identity, respectively (**Table 2**). The sequences of PCR products obtained for *sboA, spaS, ituD* and *sfp* are provided in the Supplementary Table S1.

**Table 2:** Sequence length and similarity of the antimicrobial peptides found in *Bacillus* sp. P5 and C3.

	Amplified fragment (bp)		GenBank sequence identity	
Gene	*Bacillus* sp. P5	*Bacillus* sp. C3	*Bacillus* sp. P5	*Bacillus* sp. C3
*ituD* (iturin A)	1015	NA	*B. subtilis* (AB050629.1) 99%	–
*sfp* (surfactin)	641	517	*B. amyloliquefaciens* strain JT84 (KX346253.1) 99%	*B. subtilis* strain EPC5 (HQ711610.1) 96%
*sboA* (subtilosin A)	424	603	*B. amyloliquefaciens* strain G341 (CP011686.1) 99 %	*B. thuringiensis* serovar *indiana* strain HD521 (CP010106.1) 99%
*spaS* (subtilin)	NA	323	–	*B. subtilis* (J03767.1) 100%

*NA, no fragment amplification was observed.*

### Production of Biosurfactants

The ability of strains to produce biosurfactant was first checked on blood agar plates. *Bacillus* sp. C3 and *Bacillus* sp. P5 growth resulted in clear rings of erythrocyte lysis around each colony (data not shown), which indicates the production of highly surface active compounds for both strains.

The emulsification index (E_24_) was determined for strains C3 and P5. The results showed that *Bacillus* sp. C3 emulsified only mineral oil, presenting an E_24_ of approximately 40%. *Bacillus* sp. P5 produced biosurfactants with emulsifying index of 24–57% for soybean biodiesel and toluene, respectively (**Table 3**). Emulsification of xylene was not observed.

**Table 3:** Evaluation of biosurfactant production by measuring the emulsification rate (E_24_) of different substances as hydrophobic organic phase.

	E_24_ (%)			
Fraction tested	Toluene	Xylene	Mineral oil	Soybean biodiesel
***Bacillus* sp. C3**				
Whole culture	NE^a^	NE	40 ± 0.7	NE
Supernatant	NE	NE	39 ± 0.5	NE
***Bacillus* sp. P5**				
Whole culture	59 ± 0.5	NE	40 ± 0.2	24 ± 0.0
Supernatant	57 ± 0.0	NE	42 ± 0.5	24 ± 0.0

*^a^NE, no emulsification*

The surface tension was measured in the absence of microbial cells in a surface tension meter using the digital Wilhelmy plate method. The surface tension of the culture medium decreased from 48.4 ± 2.4 mN m^-1^ (control medium) to 29.2 ± 0.2 mN m^-1^ (supernatant after growth of *Bacillus* sp. P5), suggesting the production of surfactants by the bacteria. For *Bacillus* sp. C3, the reduction of surface tension of the culture medium was not significant, with a measurement of 46.2 ± 2.6 mN m^-1^.

### Antimicrobial Activity of *Bacillus* sp. P5

**FIGURE 3:**
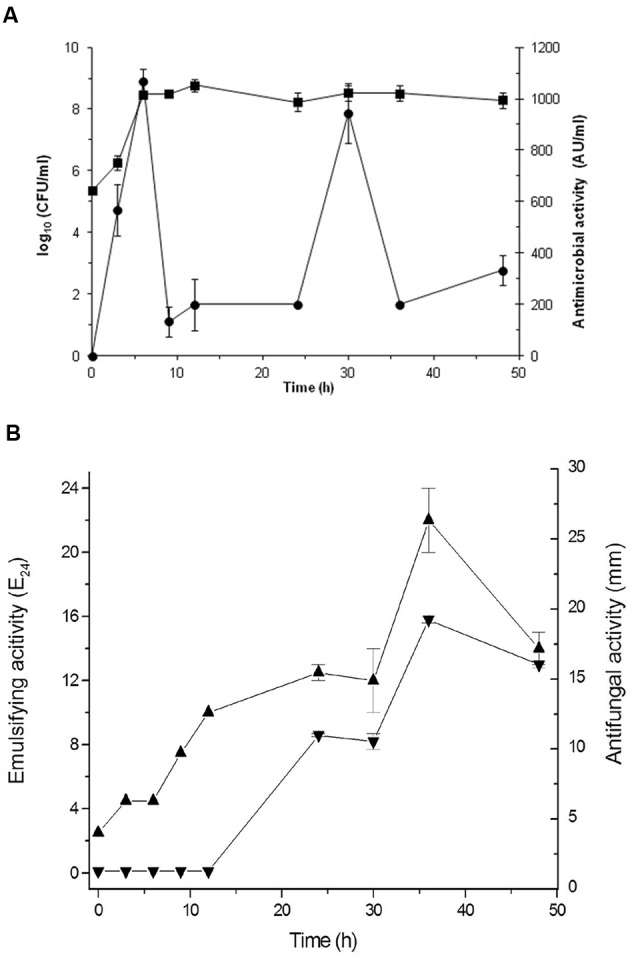
**Cultivation of *Bacillus* sp. P5 in BHI broth at 42^o^C. (A)** Cell growth (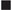) and antibacterial activity against *B. cereus* ATCC 14579 (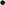) were monitored during cultivation. **(B)** Emulsification activity (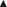, E_24_) and antifungal activity against *Fusarium oxysporum* f. sp. *lycopersici* (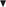) were monitored during growth. Values are the means ± SEM of three independent experiments.

Based on these results, *Bacillus* sp. P5 was selected for additional characterization. The production of antimicrobials and biosurfactants was monitored during growth of *Bacillus* sp. P5. The microorganism reached the stationary growth phase after 12 h incubation and this condition was maintained until 48 h (**Figure 3**). The initial pH of the medium was 7.2, and during the growth of *Bacillus* sp. P5, an increase in pH was observed to reach 8.7 at the end of the culture (data not shown).

The antifungal and antibacterial activities were monitored during bacterial growth. The antibacterial activity was maximal at 6 h, coinciding with the exponential growth phase, then decreasing and showing another peak at 30 h during the stationary phase (**Figure 3A**). The antifungal activity was only observed after 24 h, with maximum values at 36 h (**Figure 3B**). The emulsifying activity was produced as the cells grew, but the maximum value was reached at 36 h coinciding with the late stationary phase and the maximum antifungal activity (**Figure 3B**).

### Identification of the Active Compounds by Mass Spectrometry

**FIGURE 4:**
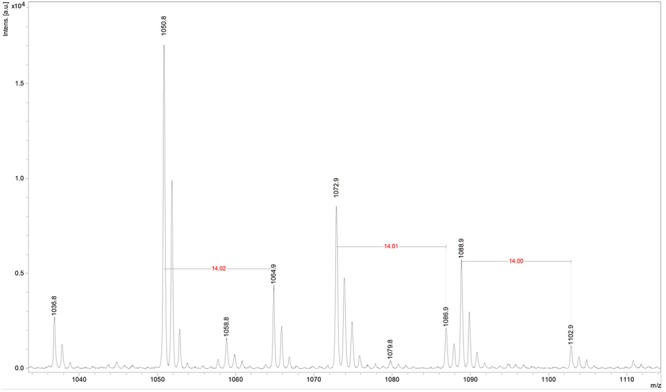
**Mass spectrum of active fraction from reversed phase HPLC of culture supernatant of *Bacillus* sp. P5.** The spectrum in the *m/z* range 1030–1110 shown data for the homologous series [M + H]^+^, [M + Na]^+^, [M + K]^+^ for surfactin A C_15_ (1036.8, 1058.8, 1074) and surfactin A C_16_ (1050.8, 1072.8, 1088.8). It also shows three additional peaks (1064.8, 1086.8, and 1102.8) that are possibly related to surfactin A C_17_ molecular species.

**FIGURE 5:**
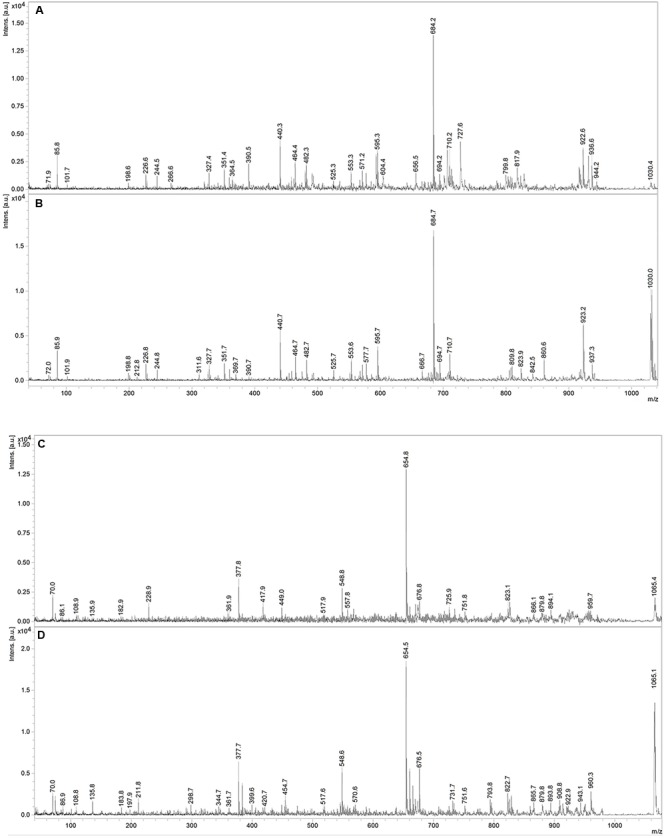
**MS/MS spectra of *m/z* 1030 peak from *Bacillus* P5 (A)** compared to a commercial surfactin standard **(B)**. MS/MS spectra of *m/z* 1065 peak obtained from *Bacillus* P5 **(C)** compared to a commercial iturin standard **(D).**

Fractions showing antimicrobial activity were subjected to MALDI-TOF mass spectrometry. Molecular masses for bacteriocins and lipopeptides were searched in the *m/z* range 1000–4000. Molecular species of lipopeptides isolated from the culture medium were found in the *m/z* range 900–1600. The typical peak for subtilosin (*m/z* 3400.57) was not observed. A selected MS spectrum of an active fraction eluted from RP-HPLC of the butanol extract of culture supernatant is shown in **Figure 4**. A peak series in the *m/z* range 1030–1110 was observed, corresponding to the [M+H]^+^, [M+Na]^+^, [M+K]^+^ adducts for SrfA C_15_ (1036.8, 1058.8, 1074.0) and SrfA C_16_ (1050.8, 1072.8, 1088.8). **Figure 4** also shows three additional peaks (*m/z* 1064.8, 1086.8 and 1102.8) that differ from a series of isoforms of SrfA C_16_ by 14 Da, a putative methylene group, suggesting a series of homolog molecules possibly related to SrfA C_17_. Comparisons of MS/MS spectra of an active fraction and commercial standards of surfactin or iturin confirm their identification. MS/MS spectra comparison of the peak *m/z* 1030 (SrfA C_13_[M + Na]^+^or Srf B C_14_ [M + Na]^+^) and peak *m/z* 1079 (ItrC_15_ [M + Na]^+^) to standards showed the same fragmentation spectra, confirming the molecular identifications (**Figure 5**). The most intense fragmentation peak of *m/z* 1030 species was *m/z* 684.7.

Analyzing the lipopeptide profiles it was observed that surfactin appears in all times tested (6, 30, and 36 h), while fengycin and iturin were detected at 6 and 36 h, respectively (**Table 4**). In addition, the lipopeptide surfactin presented more abundant isoforms during cultivation, while fengycin isoforms were detected with higher abundance at 6 h. Comparing these results with the growth curves and production of antimicrobial activity, the highest antifungal activity coincided with the time in which greater abundance of iturin lipopeptides was detected in MALDI-TOF spectrometry.

**Table 4:** Possible assignments of major *m/z* peaks detected from *Bacillus* sp. P5.

Cultivation time (h)	*m/z*	Possible assignment^a^
6	1058.5	C_15_ srfA^b^[M + Na]^+^
	1072.8	C_16_ srfA [M + Na]^+^
	1074.8	C_15_ srfA [M + K]^+^
	1485.6	C_16_ fgy^c^[M + Na]^+^
	1499.8	C_17_ fgy [M + Na]^+^ or C_15_ fgy-Val [M + Na]^+^
	1505.8	C_17_ fgy-Val [M + H]^+^
	1513.9	C_16_ fgy-Val [M + Na]^+^
	1515.8	C_17_ fgy [M + K]^+^ or C_15_ fgy-Val [M + K]^+^
	1529.7	C_16_ fgy-Val [M + K]^+^
30	994.39	C_13_ srf B [M + H]^+^
	1016.5	C_13_ srf B [M + Na]^+^
	1032.4	C_13_ srf B [M + K]^+^
	1064.7	C_17_ srf A [M + H]^+^
36	1008.4	C_13_ srf A [M + H]^+^ or C_14_ srf B [M + H]^+^
	1030.6	C_13_ srf A [M + Na]^+^ or C_14_ srf B [M + Na]^+^
	1046.6	C_13_ srf A [M + K]^+^ or C_14_ srf B [M + K]^+^
	1065.5	C_14_ itu^b^ [M + Na]^+^
	1079.5	C_15_ itu [M + Na]^+^
	1095.3	C_15_ itu [M + K]^+^
	1121.6	C_18_ itu [M + Na]^+^
	1135.6	C_19_ itu [M + Na]^+^
	1449.5	C_15_ fgy [M + H]^+^

*^a^Possible assignments based on Chen et al. (2008); Stein (2008), and Wang et al. (2004).^b^surfactin, ^c^fengycin, and ^d^iturin.*

An aqueous suspension of puba was extracted with butanol and concentrated in ZipTip^®^ C4, and molecular mass was determined by MALDI-TOF. Data were acquired in the *m/z* range 1000–4000 for the butanol extract. Two clusters were found, corresponding to iturin and fengycin. Expansion of the spectrogram of fengycin indicated protonated ion and sodium and potassium adducts, and MS/MS spectrum of precursor ion of *m/z* 1478.5 (fengycin C_17_) generated product ions of *m/z* 1079.9 and 965.8, which are compatible with fengycin A with Ala at position 6.

## Discussion

In the present study, two bacterial strains isolated from a fermented cassava product were initially identified as *Bacillus* sp. C3 and *Bacillus* sp. P5. On the basis of biochemical and physiological tests, the strain C3 was found to be closely related to species of the *B. cereus* group while strain P5 shown to be associated with the *B. subtilis/amyloliquefaciens* group. Analysis of cellular FAME identified the C3 strain as *B. thuringiensis* while P5 strain was not identified at species level. The phylogenetic analysis confirmed the allocation of strain P5 on the same cluster of *B. subtilis*, specifically with *B. amyloliquefaciens*, whereas C3 was grouped with *B. thuringiensis*. Some bacteria of the *Bacillus* genus have been detected in cassava fermented products in Brazil (Lacerda et al., 2005; Almeida et al., 2007; Santos et al., 2012) and Africa (Amoa-Awua and Jakobsen, 1995; Assanvo et al., 2006; Coulin et al., 2006; Padonou et al., 2009).

*Bacillus amyloliquefaciens* is a known producer of iturins, a family of cyclic lipopeptide antibiotics (Hiradate et al., 2002). Strains of *B. amyloliquefaciens* producing iturin have been used as biological control agents for suppressing fungal plant pathogens (Yoshida et al., 2001; Yu et al., 2002; Ongena and Jacques, 2008). *B. amyloliquefaciens* GA1 showed high inhibitory activity *in vitro* against fungi and oomycetes multiple plant pathogens, and caused a decrease in seedling disease by direct antibiosis against soil pathogens suggesting the secretion of multiple antibiotics by *B. amyloliquefaciens* GA1 (Arguelles-Arias et al., 2009). Furthermore, this same strain that had been previously identified as *Bacillus subtilis* GA1 (Toure et al., 2004) had also been implicated in reducing post-harvest infection of apples by *Botrytis cinerea*, the causal agent of gray mold.

In this work, *Bacillus* sp. P5 demonstrated inhibitory activity against various bacteria, especially those related to the genus *Bacillus* (*B. amyloliquefaciens, B. cereus*, and *B. subtilis*) as well as against two strains of *Listeria*. In addition, the inhibition of staphylococci, filamentous fungi and the yeast *C. tropicalis* was also observed. Motta (2006) found a broad inhibitory spectrum by an isolate of *Bacillus* sp., including several strains of the genus *Bacillus* and various strains of *Listeria* spp. Similar results were found for *B. cereus* 8A, producing a bacteriocin that inhibits *L. monocytogenes, Clostridium perfringens, Streptococcus bovis, Micrococcus luteus*, and several species of *Bacillus* (Bizani and Brandelli, 2002). The susceptibility observed by *L. monocytogenes* suggests the high effectiveness of the lipopeptides produced by *Bacillus* sp. P5 against this important pathogen, whereas *Bacillus* sp. C3 inhibited only few microorganisms.

The genes *spaS* and *sboA*, related to the antimicrobial peptides subtilin and subtilosin A, respectively, were detected in *Bacillus* sp. C3. The bacteriocins subtilin and subtilosin A were formerly identified from *B. subtilis* ATCC 6633 and *B. subtilis* 168, respectively (Babasaki et al., 1985; Banerjee and Hansen, 1988; Chung et al., 1992). The detection of genes for subtilin and subtilosin A was not previously reported in species belonging to the *B. cereus/thuringiensis* group.

For *Bacillus* sp. P5 the genes *sfp, sboA*, and *ituD* related to production of antimicrobial peptides surfactin, subtilosin A, and iturin A, respectively, were found *Bacillus* spp. may produce a variety of antimicrobial peptides, and their synthesis is under a complex regulation influenced by environmental conditions and the presence of competing organisms (Stein, 2005; Benitez et al., 2011). The co-production of lipopeptides by a strain could be advantageous, since a synergistic effect may occur. The simultaneous production of substances such as iturin A and surfactin has been reported for *B. subtilis* (Ahimou et al., 2000) and possibly for *B. amyloliquefaciens* (Souto et al., 2004), whereas co-production of surfactin and bacilomycin has been reported for *B. subtilis* (Zhang et al., 2008). However, the co-production of three or more lipopeptide antibiotics is unusual, as described by Kim et al. (2010), who demonstrated the production of iturin A, fengycin A, and surfactin by *B. subtilis* CMB32. The broad inhibitory spectrum of strain P5 suggests that diverse antimicrobial molecules could be produced.

Different isoforms of surfactin have been detected in this work and they exhibited variation in the length of the β-hydroxy-fatty acid from 13 to 17 carbons units. The protonated precursor ions *m/z* 1036.8, 1050.8, and 1064.9 may be assigned as surfactin homologs with 15, 16, and 17 carbon β-hydroxy-fatty acid moiety, respectively. Adducts of sodium and potassium were also observed. These surfactin isoforms have been previously described (Hue et al., 2001; Vater et al., 2002).

MS/MS fragmentation of the molecular specie *m/z* 1030 of an active fraction obtained by HPLC of the butanol extract of a culture supernatant was compared to the fragmentation of surfactin standard, and it shows many similarities. In both spectra the most intense peak has *m/z* 684.7. This fragment ion can be used as a characteristic marker for surfactin homologs (Hue et al., 2001; Vater et al., 2002). Expansion of the spectrogram of fengycin indicated protonated ion and sodium and potassium adducts and MS/MS spectrum of precursor ion of *m/z* 1478.5 (fengycin C_17_) generated product ions of *m/z* 1079.9 and 965.8, which are considered fingerprints of fengycin A Ala at position 6, as previously described Wang et al. (2004).

MALDI-TOF is shown to be an efficient tool for identification of antimicrobial peptides in the range from 1 to 5 kDa (Stein, 2008). Analysis by mass spectrometry of the lipopeptides from *Bacillus* sp. P5 showed characteristic peaks for surfactin, iturin, and fengycin isoforms, including ions corresponding to [M + Na]^+^ and [M + K]^+^ adducts. Some compounds produced by *Bacillus* sp. P5, under these culture conditions, presented a mass difference of 14 Da, which corresponds to the molecular weight of one CH_2_ group. This corresponds to different isoforms for each lipopeptide, which vary in the chain length of their fatty acid components. The presence of sodium and potassium adducts also favor differences of 22 or 38 Da, respectively, in the peaks. These results suggest that the strain P5 produces a diversity of surfactin, iturin, and fengycin isoforms that may be associated with its antimicrobial activity and surfactant properties. Vater et al. (2002) used an innovative method for rapid and sensitive detection and efficient structural characterization of lipopeptide biosurfactants by MALDI-TOF mass spectrometry, and revealed three lipopeptide complexes: surfactins, iturins, and fengycins. These same lipopeptides were produced by *Bacillus* P5 strain.

*Bacillus* sp. C3 and P5 showed hemolysis on sheep blood agar, and *Bacillus* sp. P5 presented emulsifying indexes of 57 and 40% for toluene and soybean oil, respectively. These values are considered high as compared with other values found in the literature (Bodour and Maier, 2002; Youssef et al., 2004), whereas *Bacillus* sp. C3 only present emulsifying activity on mineral oil. Although the method of Hsieh et al. (2004) was developed to investigate surfactin among species related to *B. subtilis*, lysis of blood erythrocytes can be related to production of other active compounds besides surfactin. Bueno (2008) tested eight bacteria belonging to the genus *Bacillus* to produce emulsifiers that decrease the surface tension at least 20%, whose emulsifying index was stable after 24 h. Different *Bacillus* strains, including *B. subtilis, B. licheniformis* and *Bacillus* sp. showed effective emulsification ranging from 43 to 48% for mineral oil and 20–45% for soybean oil (Velho et al., 2011).

Natural biosurfactants have an advantage over the synthetic ones, because most of them are biodegradable and generally less toxic than conventional surfactants (Singh and Cameotra, 2004). The strain *Bacillus* sp. P5 caused hemolysis on blood agar and showed elevated emulsifying activity on mineral oil and soybean oil, and reduction of the surface tension of the culture medium to levels considered excellent for biosurfactants (Willumsen and Karlson, 1996). Different nutritional conditions are required for the production of these compounds, predisposing the production of one or the other (Thaniyavarn et al., 2003).

The significant reduction of surface tension by culture supernatants of *Bacillus* sp. P5 agrees with the production of surfactin. Surfactin represents one of the most effective surfactant studied so far, capable of reducing the surface tension of water from 72 to 27.9 mJ m^-2^ at a concentration of 0.05% (Cooper et al., 1981). Similar result to that observed in this work was described for *B. licheniformis* BAS50, which produce surfactants that reduce the water surface tension to values close to 29 mN m^-1^, still presenting stability in salinities above 40% (Yakimov et al., 1995). *B. licheniformis* JF-2 also produced biosurfactants that reduced the surface tension of water to values below 27 mN m^-1^ (Javaheri et al., 1985).

## Conclusion

This study identified a new bacterial isolate named *Bacillus* sp. P5, closely related with the *B. amyloliquefaciens* species, which carries genes for iturin A, surfactin and subtilosin A production, and another new isolate named *Bacillus* sp. C3, that was shown to be related with the *B. thuringiensis* species, which carries subtilosin A and subtilin genes. *Bacillus* sp. P5 showed activity against pathogenic fungi, pathogenic bacteria and bacteria that causes food spoilage in industry, as *L. monocytogenes* and *B. cereus*. *Bacillus* sp. C3 showed minor action against the microorganisms tested, although reduction in sporulation in fungi like *A. flavus* and *A. niger* was observed, as well as partial inhibition against bacteria belonging to the *B. cereus* group. Considering that *puba* is often manufactured under poor hygienic conditions, production of antimicrobial substances like iturin, surfactin and fengycin by autochthonous *Bacillus* strains is probably important for the safety of this product and further studies are needed to evidence the exact role of these antimicrobial substances in this food.

## Author Contributions

KP contributed to the development of experimental research, data analysis, and preparation of the article. JV contributes to the development of experimental research. FL contributed in detection of antifungal activity and phylogenetic characterization. JP contributed to the phylogenetic analysis. DS contributed to performing, and analyzing of the active compounds by mass spectrometry. JO contributed to the performance of reversed phase HPLC. RV analyzed the bioinformatics data. SC isolated, and contributes to the presumptive identification of *Bacillus* strains from puba. JN, AB, and RN contributed to the assisted in the design of the work, assisted in critical data interpretation, and in preparation of the article. All authors have participated in this study and commented on the manuscript.

## Conflict of Interest Statement

The authors declare that the research was conducted in the absence of any commercial or financial relationships that could be construed as a potential conflict of interest.

## Footnotes

1 www.ncbi.nlm.nih.gov/BLAST

2 www.ncbi.nlm.nih.gov/BLAST

## Supplementary Material

FIGURE S1 **Parasporal crystals.** Ellipsoidal subterminal spores are phase-bright and parasporal crystals are less phase-bright than the spores.
