# Supplementary figures and images for: Bacillus spp. Isolated from Puba as a Source of Biosurfactants and Antimicrobial Lipopeptides
Source: Front Microbiol. 2017 Jan 31;8:61. doi: 10.3389/fmicb.2017.00061 (PMC5281586; doi:10.3389/fmicb.2017.00061)

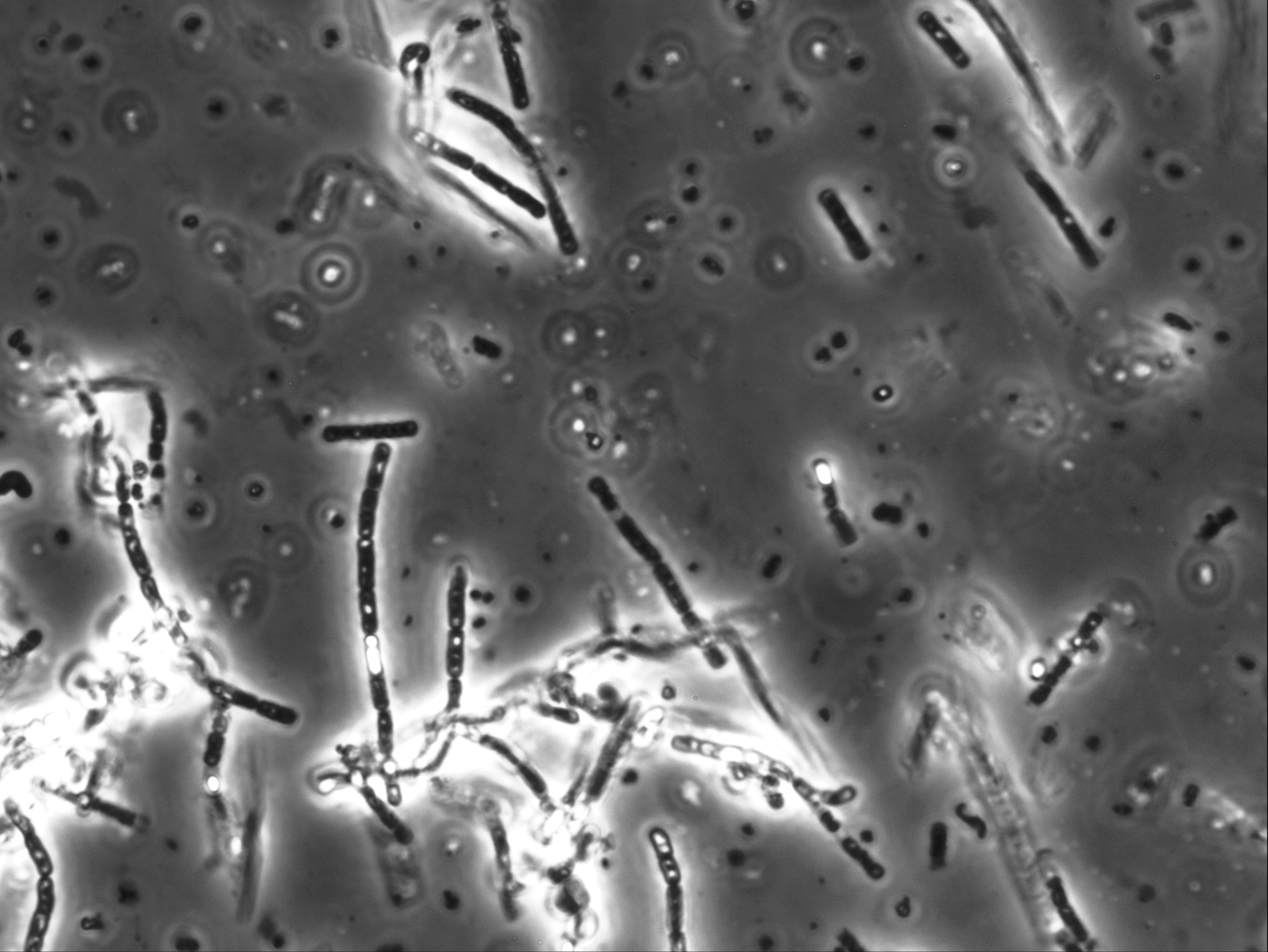

Supplement: FIGURE S1 — Parasporal crystals. Ellipsoidal subterminal spores are phase-bright and parasporal crystals are less phase-bright than the spores. [file Image_1.jpeg]
