# Supplementary material for: Bacillus spp. Isolated from Puba as a Source of Biosurfactants and Antimicrobial Lipopeptides
Source: Front Microbiol. 2017 Jan 31;8:61. doi: 10.3389/fmicb.2017.00061 (PMC5281586; doi:10.3389/fmicb.2017.00061)
Supplement: Supplementary file 2 [file Table_1.docx]

**Supplementary Material**

**Table S1**. Nucleotide sequences of PCR products of *spaS*, *sboA*, *sfp* and *ituD* from *Bacillus* strains C3 and P5.

| **Strain / gene** | **Nucleotide sequence** |
| --- | --- |
| C3 / *spaS* | TTAGGGGTATTAAACATGGAATTAGGCTCAAAAACAGATTGGACAAAAGCATTATTAATTTAATAAAAAAAGGAAAAAAATGATAAAATCTTGATATTTGTCTGTTACTATTTAGGTATTGAAAGGAGGTGACCAATATGTCAAAGTTCGATGATTTCGATTTGGATGTTGTGAAAGTCTCTAAACAAGACTCAAAAATCACTCCGCAATGGAAAAGTGAATCACTTTGTACACCAGGATGTGTAACTGGTGCATTGCAAACTTGCTTCCTTCAAACACTAACTTGTAACTGCAAAATCTCTAAATAAGTAAAACCATTAGC |
| C3 / *sfp* | ATGAACGGTTCATGTCTTTCATATCGCCTGAAAAACNGGAGAAATGCCGGAGATTTTATCATAAAGAAGATGCTCACCGCACCCTGCTAGGAGATGTGCTCGTTCGCTCGGTTATTAGCAGGCAGTATCAGCTGGACAAAGCTGACATCCGCTTCAGCGCGCAGGAATACGGGAAGCCTTGCATCCCTGATCTTCCTGACGCCCATTTCAACATTTCTCACTCCGGCCGCTGGGTCATTTGTGCGTTTGATTCACACCCGATCGGCATCGATATTGAAAAAATGAAACCGATCAGCCTTGAGATCGCCAAACGTTTCTTTTCAAAAACAGAGTACAGCGACCTTTTAGCAAAAAACAAGGACGAGCAGACAGACTATTTTTATCATCTATGGTCAATGAAAGAAAGCTTTATCAAACAGGAAGGCAAAGGCTTATCACTTCCGCTAGATTCCTTTTCAGTGCGCCTGCATCAGGACGGACAAGTATCCATTGAGCTTCCGGACAGCCATACACCATG |
| C3 / *sboA* | AGCATTTCCTTCTTTAATTGAAGCGAAATACGTATACTGTACAGCAAGCATACCGAATAGACCAAACAAAATCATTTTAATAGCATCAGATTTTTGTTTCCAAATGCTAAATATCTCTTTTTTTCTCGTTCCGAATGAGGAAATAATGAGCAAAATAATTCCAGATATAAGTAAACGGATCGTAATTAACCATTCGGTTGAAACATTGTCATATTGAAAAAGTTGTTGTGCAGCTGTACCAGACAAGCCCCATAAACAAGCACCGATTATGACCATTATAATTCCTTTTAATCGATTTGAATCCATATGATCTAAAATTCTCCTTTCTAAAAATACAAATTGAATACAATATGCAATATATCACAGAATGACTCATTAATAAACTGAAATTTCTAATTTGTTTTTTATGTATAGTAAAATATTGTAGAGGAGGGGGAGTATATGTTAGCTGGATTTTTAGTTTGTTTATTTGTTGGGTTACTTATCATTTTTTTAGGGTATCAAATACATGTGAAAAAGAGGGTGTTTTTGTTAGCAGGATATCAAGAAGAAACGTTTGTTGGAGATAAAAATAAATTAGCGAAGCTTTCAGGATCATTTTCT |
| P5 / *ituD* | ATGGGCAAACAATTTTGGAATGATTTTGTGCTCGCAAAGAGATTGTTTGAAGAAGCGAGCGATGCGATCTCCTTGGATGTAAAAAAACTGTGTTTTAACGGAGATATGAATGAATTGACAAAGACAATGAACGCGCAGCCCGCTATTTTAACGGTCAGTGTGATTGCTTTTCAAGTGTATATGCAGGAAATAGGGGTGGAGCCCCGCTTCCTGGCAGGCCATAGCTTAGGCGAATATTCAGCGCTTGTCTGTGCCGGCGCCCTTTCTTTTCAGGATGCCGTTACACTTGTAAGGGAGCGGGGAATTCTTATGCAAAATGCGGATCCCCAGCAGCAGGGGACGATGGCCGCCGTGACACAGCTCTCTCTCCAAACGTTGCAGGAAATATGTTCGAAAGTATCGACGGAAAACTTTCCGGCAGGTGTAGCCTGCATGAATTCAGAACAGCAGCATGTGATTTCCGGACACCGGCAAGCTGTGGAACGTGTCATCAAGATGGCGGAGGAAAAGGGTGCGGCATACACTTATTTGAATGTCAGTGCGCCTTTTCACAGTTCGATGATACGATCAGCCTCCGAACAATTCCAGACTGTTTTACACCGGTATTCCTTCCGAGATGCCGCATGGCCGATCATTTCAAATGTCACCGCGCGCCCTTACAGCAGCGGAAATTCGATCAGCGAACATCTCAAGCAGCACATGACGATGCCGGTTAGATGGACAGAATCGATGCATTACTTGCTTTTACATGGAGTCACAGAAGTCATCGAAATGGGTCCGAACAATGTCTTAGCCGGTCTGCTGAGAAAAACAACGAATCACATTGTACCTTATCCCTTAGGACAGACATCTGATGTTCCCCCGCTTTCCAATTCAACGGAAAGAAAGAAACATATTGTCCATTTACGCAAAAAACAACTGAATAAATTGATGATTCAATCCGTCATTGCGCGAAATTACAACAAGGATTCAGCGGCTTATTCCACTATGACGACGCCATTATTTACGCAAATCC |
| P5 / *sboA* | TCATCCTCGTCACAGACTTCACATGGAGTGTTATCGGTGTCTCTTGCTTCATTTTGACACGTCCTTTTCATCATTCCACTTTGACTTGAATAGTATAATCATTCAGCTTGTAAACTTCAACTGCTTCTATCTTACCATCATTGCTCATTAAATTTGAAGATAAACCTCATAAAAAGCAGTTCCTTATGAGAAGAGAAAACCATATCACTATGTCACCCAATCCGTAAAAAACATGACAGCAGCTTTTCAGAAACTCACACAGTCTCATCGAAGCTGCTGCGACTTCCAGCAACATAGATTGTGCAGCAGAAATGCCAACGATGTCAAAGCGCTGCGTGACAGCTATAAAAACGTCCGCCAAACACACGTGATTGCTGCTGTATTATGCCAAGTCATCATATTCGGCTGCGTGTTAGAAATCGAT |
| P5 / *sfp* | TATATGGACCGCCCGCTTTCTGCAGGGGAAGAGGATCGGATGATGGCGGCCGTGTCCGCCGAAAAGCGGGAAAAATGCCGACGCTTTTACCATAAGGAGGATGCTCCCGCACCTTGATCGGCGACATGCTGATCCGCACCGCTGCGGCGAAGGCTTACGGACTTGATCCGGCCGGGATTTCATTCAGCGTTCAGGAATACGGAAAGCCGTACATCCCCGCGCTTCCGGACATGCACTTTAACATTTCCCACTCCGGTCGCTGGATCGTGTGCGCCGTTGATTCAAAACCGATCGGCATTGATATTGAAAAAATGAAGCCCGGCACGATTGATATCGCCAAACGGTTTTTTTCGCCGACGGAATACAGTGATCTGCAAGCGAAACACCCCGATCAGCAGACCGATTATTTTTACCACCTGTGGTCGATGAAAGAAAGCTTTATCAAGCAGGCCGGAAAAGGGCTTTCCCTGCCGCTTGATTCATTCAGCGTCCGCCTCAAAGACGACGGCCATGTGTCCATTGAGCTTCCGGACGGGCATGAACCTTGTTTCATCCGCACATATGATGCGGACGAGGAGTATAAGCTGGCCGTTTGTGCGGCGCATCCCGATTTTTGTGACGGGATTGAGATGAAAACGTAC |
